# Min oscillations in bacteria as real-time reporter of environmental challenges at the single-cell level

**DOI:** 10.1098/rsob.230020

**Published:** 2023-07-26

**Authors:** Ingrid V. Ortega, Felipe Viela, Cristina Flors

**Affiliations:** ^1^ Madrid Institute for Advanced Studies in Nanoscience (IMDEA Nanociencia), C/ Faraday 9, Madrid 28049, Spain; ^2^ Unidad Asociada en Nanobiotecnología, Centro Nacional de Biotecnología (CNB-CSIC-IMDEA), C/ Faraday 9, Madrid 28049, Spain

**Keywords:** fluorescence microscopy, fluorescent reporters, Min oscillations, single cell imaging, sublethal, bacterial stress response

## Abstract

Min oscillations are a fascinating mechanism used by *Escherichia coli* to find their middle. Beyond their biological role, they provide a convenient and relatively unexplored method to monitor the effect of sublethal environmental challenges on bacterial physiology in real-time and at the single-cell level. In this review, we discuss the original papers that put forward the idea of using Min oscillations as a reporting tool to monitor the effect of extracellular cationic compounds, including antibiotics. More recent work from our laboratory explores this tool to follow bacterial response to other challenges such as weak mechanical interactions with nanomaterials or photodynamic treatment. We discuss the physiological meaning of the changes in Min oscillation period, likely related to membrane potential dynamics, as well as the benefits and limitations of using oscillations as a reporter in fluorescence microscopy. Overall, Min oscillations are a useful addition to the fluorescence microscopy toolbox in order to visualize stress responses in *E. coli*, and have the potential to provide full mechanistic understanding of the events that lead to bacterial cell death in different contexts.

## Graphical abstract

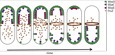

## 1. Introduction

One of the most intriguing questions in microbiology is how bacteria find their middle in order to properly divide [1]. Decades of research have established that the Min protein system in *Escherichia coli*, which oscillates from pole to pole of the cell, constitutes a spatio-temporal regulatory mechanism for positioning the division machinery [1,2]. Unravelling the interplay between the components of the Min system has led to advances in understanding self-organized pattern formation *in vivo* and *in vitro* [3–5]. In addition to cell division, Min proteins have been reported to play a role in other bacterial functions such as motility, colonization and virulence [6]. Beyond its biological role, the oscillatory behaviour of the Min system has been used in fluorescence microscopy as a tool to report on the physiological state on *E. coli*, as it has been shown to be sensitive to a range of environmental challenges [7–12]. We review herein the use of Min oscillations as a single-cell reporter for sublethal treatment with antibiotics, temperature, mechanical interaction, or light-induced generation of reactive oxygen species (ROS). We discuss the physiological meaning of changes in Min oscillation behaviour, probably related to membrane potential dynamics [13], as well as the advantages and limitations of monitoring oscillations in quantitative fluorescence microscopy.

## 2. Biological role of the Min system

The spatial location of the bacterial division septum is of primary importance for the correct bacterial duplication [14,15]. In *E. coli*, the Min system is responsible for the placement at the midcell of the Z-ring [16], a supramolecular structure of polymerized FtsZ that recruits other proteins forming the divisome, a complex that mediates cytokinesis [17,18]. The molecular interplay between the proteins in the Min system results in a tight control and precise location of the Z-ring formation [14].

**Figure 1.**
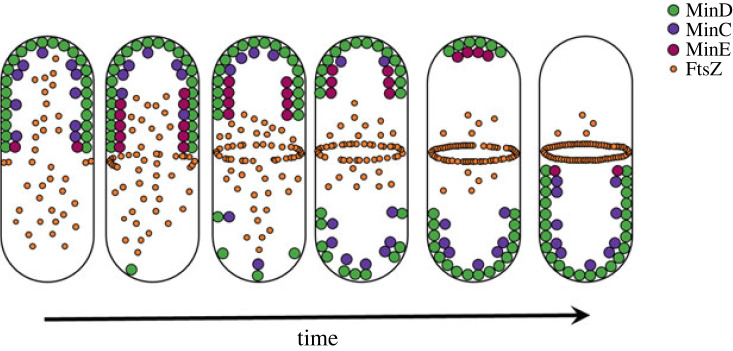
Interplay of the proteins that compose the Min system to drive the localization of the division machinery.

The Min (or MinCDE) system consists of three proteins, MinC, MinD and MinE, with highly dynamic interactions (figure 1) [3,4,19]. MinD is a monomeric ATPase that cooperatively self-assembles on the cytoplasmic side of the inner bacterial membrane upon ATP binding, interacting with membrane phospholipids in the presence of Mg^2+^ and covering one cell pole up to the midcell [20]. MinD harbours a membrane-targeting sequence (an amphipathic helix at the C-terminus) with a weak affinity, and therefore only supports membrane binding in the presence of more than one copy [4,21,22]. At a threshold concentration of membrane-bound MinD, MinC is recruited. MinC is then displaced by MinE, as they compete for the same binding site in MinD [20,23]. Upon interaction with MinD, MinE changes conformation and also binds to the membrane [24,25]. MinE stimulates the ATPase activity of MinD by inducing a conformational change in a region involved in ATP binding [20], resulting in the monomerization and detachment of MinD from the bacterial inner membrane at one of the cell poles, and increasing the cytoplasmic concentration of MinD and MinC. Meanwhile the process starts at the opposite pole, and this reaction–diffusion mechanism of membrane attachment and detachment is responsible for the apparent pole-to-pole oscillations [26]. MinC, which is an inhibitor of FtsZ assembly, does not participate in the reaction–diffusion mechanism and can be considered an effector of the system [4]. The interactions between MinD/E proteins and the differences in their diffusion rates creates an intracellular gradient of soluble MinD and MinC that inhibits the polymerization of the Z-ring at the cell poles and enables its formation at the cell centre [27,28].

While the Min system has been extensively studied, the exact interaction mechanisms are not yet fully understood at the structural level. We point the reader to comprehensive reviews that discuss in detail the interplay between its components, and how their self-organization leads to pattern formation [1–4,15,29,30]. Moreover, other studies have suggested that the Min system participates in other cellular processes beyond cell division such as motility, colonization, virulence and RNA decay [6].

## 3. Min oscillations as reporter of environmental challenges

Fluorescent protein fusions with MinD and time-lapse microscopy have been used extensively to monitor its oscillation dynamics [31], although Min proteins are sensitive to fusions and it is important to verify that their function is not impaired [32]. The oscillation period depends on several parameters, and ranges between 40 and 120 s [4]. There are some inherent features that contribute to this variability, such as the type of strain, or MinD/MinE ratios [31]. However, it has been shown that external factors such as temperature or sublethal doses of antibiotic compounds also affect the oscillation period. On that basis, the period of Min oscillations was proposed as a reporter of bacterial physiological state. In this section, we describe the effect on oscillations by a range of different environmental challenges.

### 3.1. Temperature

**Figure 2.**
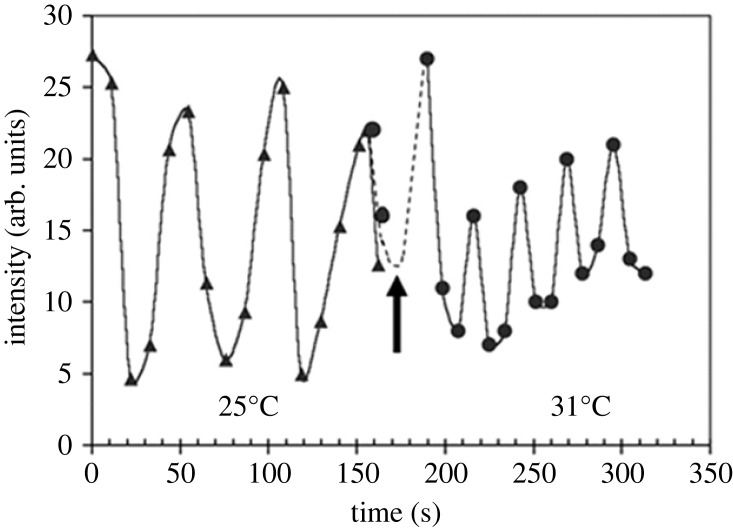
Temperature dependence of Min oscillations in *E. coli*. A 6°C jump rapidly changes the oscillation period from 51 s to about 26 s. Adapted with permission from Touhami *et al*. [11].

The temperature dependence of the Min system oscillation period was an early report of the effect of external factors on Min dynamics [11]. The study was motivated by the observation that the growth rate in *E. coli* increases four-fold between 21°C and 37°C according to an Arrhenius law. It was found that Min system oscillations follow the same model and are four times faster at 40°C than at 20°C. The authors also reported that the response to temperature changes is very fast, and a 6°C jump decreases the oscillation period from 51 s to 26 s within a few seconds (figure 2). The molecular origin of this phenomenon was suggested to be related to temperature-dependent rates of ATP hydrolysis, followed by nucleotide exchange, which govern the interactions between proteins in the Min system or with other cellular components, and were previously shown to be the rate-limiting factors for Min oscillation [33]. A subsequent model based on experimental data provided additional evidence to support this hypothesis as an explanation for the observed temperature dependence [34].

From the practical point of view, the strong temperature dependence of this phenomenon introduces a large variability in the oscillation periods when experiments are performed at (varying) ambient temperatures, and therefore temperature control should be used to achieve best reproducibility.

### 3.2. Polycations and cationic antimicrobial compounds

Another early study focused on using Min oscillations as an intracellular reporter to study the bacterial response to divalent cations, such as Ca^2+^ and Mg^2+^, which are necessary for basic functions, as well as to polycationic antimicrobial compounds [10]. At a time when genetically encoded cation sensors were still in their infancy, this strategy provided an interesting possibility to follow the effect of polycation cytoplasmic penetration in real time and at the single cell level.

**Figure 3.**
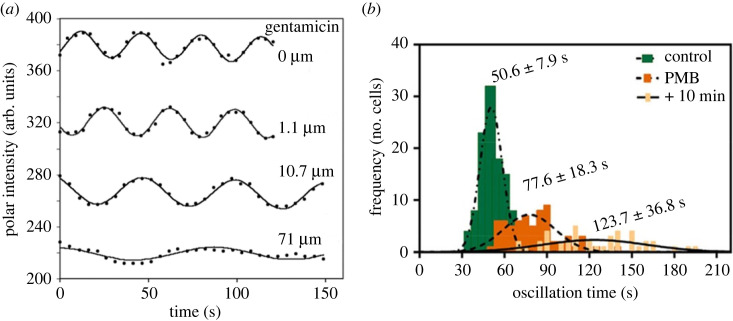
(*a*) Effect of the antibiotic gentamicin on the Min oscillation period of a single *E. coli* PB103 cell (from reference [10]). (*b*) Histograms showing the increase in pole-to-pole oscillation period induced by addition and 10 min incubation with polymyxin B (PMB) in *E. coli* DH10*β* cells (from Ortega *et al*. [8]).

Increasing concentrations of Ca^2+^, Mg^2+^, the antimicrobial peptide protamine and the aminoglycoside gentamicin increased the average Min oscillation period in a concentration-dependent manner (figure 3*a*). Interestingly, the effect was transient for divalent cations, even with constant extracellular concentration, consistent with homeostasis of intracellular Ca^2+^. On the other hand, the increase of the oscillation period was irreversible for the antimicrobial compounds. Moreover, moderate concentrations of Ca^2+^ or Mg^2+^ reduced the effect of the antimicrobial agents on Min oscillations, suggesting that they prevented the latter from entering the cell [10].

Slower Min oscillations were also observed upon treatment with polymyxin B (PMB), a cationic peptide with antimicrobial activity that is specific towards Gram-negative bacteria and permeabilizes both the outer and the cytoplasmic membranes [35,36]. Addition of PMB slowed down Min oscillations and increased cell-to-cell variability in the oscillation period (figure 3*b*) [7,8]. By contrast to gentamicin and protamine, the effect was independent of concentration, suggesting that ionic effects dominate over structure-specific mechanisms above a certain threshold of PMB concentration [7]. Unexpectedly, treatment with the related compound polymyxyn B nonapeptide (PMBN) which lacks a fatty acid present in PMB and only disrupts the outer membrane, speeds up oscillations [7]. This observation was unusual, since the entire Min protein system exists in the cytoplasmic space of the cell. Moreover, this is to the best of our knowledge the only example of an extrinsic factor, besides increasing temperature, which shortens the Min oscillation period.

The increase in the Min oscillation period observed for polycations was used to report the effect of poly L-lysine (PLL) [12], a cationic polymer with well-established antimicrobial action but that is commonly used to immobilize bacterial cells for microscopy experiments. The effect of different PLL preparations on bacteria was compared by following their Min oscillation period. This study highlighted that oscillations in bacteria immobilized on thick PLL coatings slowed down significantly, and therefore these substrates have a significant effect on bacterial physiology and should be avoided. On the other hand, in milder immobilization conditions pole-to-pole oscillations were only moderately slowed down and stable for hours. This constitutes a convenient method to optimize bacterial immobilization conditions for live-cell microscopy experiments [8,9].

### 3.3. Effect of reactive oxygen species: phototoxicity and photodynamic treatment

Photosensitized formation of ROS, such as singlet oxygen, superoxide anion, hydrogen peroxide or hydroxyl radical, is part of the most common mechanism of fluorophore-induced phototoxicity in fluorescence microscopy experiments. Early studies found that cumulative light excitation slowed down GFP-MinD oscillations by about 10 s [10], presumably due to light-induced generation of ROS by GFP [37] or buffer components [38], and therefore providing a sensitive tool to monitor phototoxicity in bacteria.

**Figure 4.**
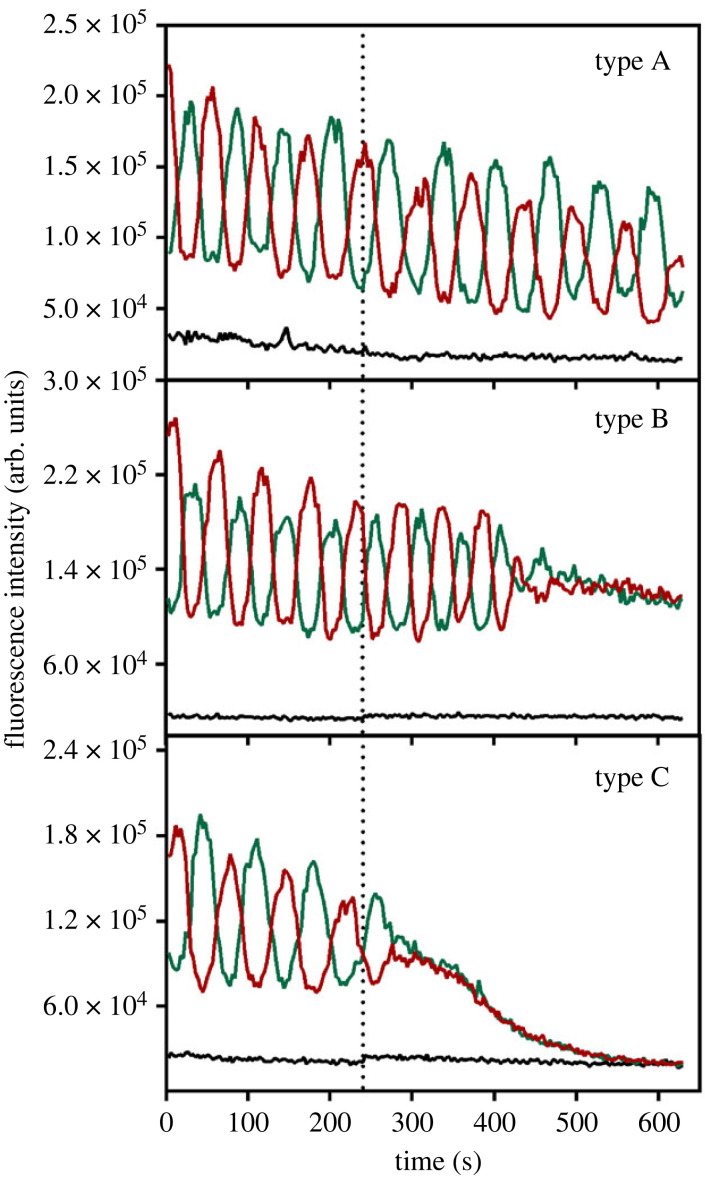
The MinD oscillation pattern in individual *E. coli* DH10*β* cells is sensitive to photodynamic treatment with increasing concentrations of the photosensitizer methylene blue (MB). At 0.5 µM MB, 41% and 59% of the cells show a type A and B pattern, respectively. At 1 µM MB, type B behaviour increases to 92%. At 2 µM MB all studied cells show a type C pattern, which corresponds to major cell membrane damage and leaching out of GFP-MinD. The black curves correspond to the fluorescence background values. The dotted line shows the start of red-light illumination. From Ortega *et al*. [8].

The interaction between bacteria and ROS is also important in the context of photodynamic inactivation (PDI), a strategy that has emerged as a response to the increasing threat of multidrug resistance [39]. PDI combines light, a photosensitizer and molecular oxygen in order to generate cytotoxic ROS that oxidize lipids, proteins, DNA and other biomolecules. While PDI is an increasingly popular method, questions remain about its mechanism and the inflicted functional changes [40,41]. In this context, our laboratory has recently explored the effect of sublethal photodynamic treatment of *E. coli* on MinD oscillations [8], since phototoxicity was suggested to affect the oscillation period as described above. We found that irradiation of bacteria in the presence of the photosensitizer methylene blue disrupts the MinD oscillation pattern depending on its concentration. This method is sensitive enough to distinguish the effect of the three photosensitizer concentrations tested in irradiation conditions (figure 4). While not discussed in the original paper, an interesting observation is that, in contrast to the phototoxic effects observed in cumulative irradiation of *E. coli* expressing GFP-MinD, which slow down the oscillation [10], photodynamic treatment results in an abrupt interruption, reflecting divergent physiological consequences of ROS. Similarly to the effects on Min oscillations of the antibiotics PMB and PMBN described in the previous section, it is likely that the difference stems from the location of the photosensitizer: cytoplasmic for GFP-MinD and outside the cytoplasm for methylene blue. The sensitivity of the Min oscillation pattern to sublethal ROS challenges from both PDI and fluorophore-induced phototoxicity is therefore a useful reporting tool for mechanistic studies of the consequences of photosensitized ROS formation in bacteria.

### 3.4. Mechanical interaction

**Figure 5.**
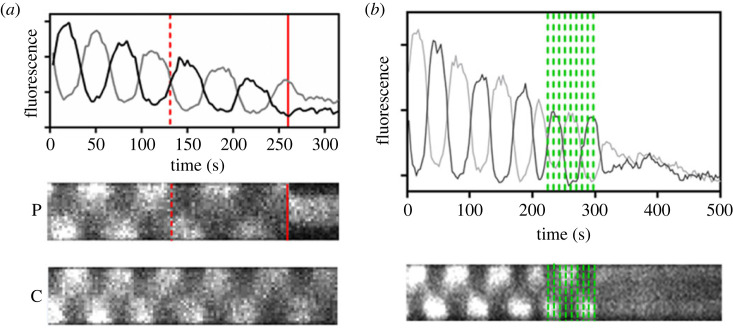
Simultaneous AFM nanoindentation and fluorescence imaging of Min oscillations in *E. coli* DH10*β*. (*a*) Pole-to-pole oscillations of GFP-MinD upon indentation of an individual bacterium with a low force (5 nN, dotted lines) and above critical damage of the cell wall (45 nN, solid lines). Black and gray curves correspond to the average fluorescence intensity of each pole. P and C show kymographs of the punctured and a control (nonindented) bacterium, respectively. The dimension of the *y*-axis in both kymographs is about 2.5 *μ*m. The gradual decrease in fluorescence intensity in all panels is due to GFP photobleaching. (*b*) Pole-to-pole oscillations of GFP-MinD upon repeated indentation (5 nN for each green line). The dimension of the *y*-axis in the kymograph below is about 3 *μ*m [9,42].

Min oscillations have also been used to monitor the effect of mechanical damage on the physiological response of bacteria, in the context of understanding the interaction between bacteria and ‘mechano-bactericidal’ nanomaterials [9]. Experiments were first performed by nanoindenting an *E. coli* cell with the tip of an atomic force microscope (AFM) and simultaneously monitoring its Min oscillations by fluorescence microscopy. Figure 5*a* shows the oscillatory behaviour of the Min system in an individual bacterium expressing GFP-MinD. Before puncture, the oscillation period is about 63 s. After one indentation with a low force of 5 nN (dotted line), below the rupture threshold of the cell wall of about 20 nN [9], the oscillation slows down only slightly. However, indentation with a force above the threshold (45 nN, solid line), which typically leads to cell wall damage and positive propidium iodide staining, results in the abrupt halt of the oscillation. Interestingly, the repeated application of low forces of 5 nN also results in the halt of Min oscillations (figure 5*b*), but no propidium iodide staining, which we interpreted as bacteria being compromised by a fatigue effect [9], i.e. stress responses that lead to high levels of ROS [43,44], impaired metabolic activity [45] or altered genomic or proteomic profiles [46]. Thus, these changes may eventually lead to cell death with no direct cell wall rupture. The observed fatigue effect is consistent with the suggestion that the antibacterial properties of high aspect ratio colloidal nanoparticles such as single-walled carbon nanotubes (SWCNTs) may stem from the accumulative action of many low force collisions [47]. This study also highlighted that cell wall integrity markers do not provide a complete picture of bacterial viability, and that using Min oscillation as a reporter for bacterial physiological state is a useful complement to study the events that lead to cell death.

**Figure 6.**
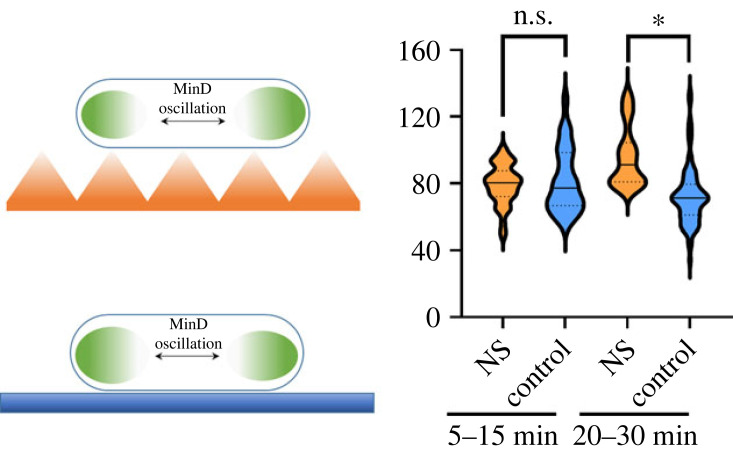
Impact of polymer nanotopographies on bacterial physiology. Violin plot showing the distribution of MinD oscillation data at 2 different time ranges on the nanostructured topography (NS, orange) compared to a flat surface (control, blue). Solid line represents mean value, dashed lines represent 1st and 3rd quartile (*p < 0.05). Adapted from Viela *et al*. [48].

A subsequent study has investigated how Min oscillations in *E. coli* respond to weak mechanical perturbations in a more realistic scenario of interactions between bacteria and mechano-bactericidal nanomaterials [48]. To that end, nanostructured topographies and ‘nanodarts’, exemplified by flowing SWCNTs, were used to inflict a physiological response on bacteria. In both cases, Min oscillations slowed down, and it was estimated that the contact time at which the initial stages of bacterial death occur is in the order of a few tens of minutes (figure 6).

## 4. What are Min oscillations reporting?

While initial reports were unclear about the specific effect(s) of external factors on the oscillation period of the Min system [10,12], it was later shown that MinD oscillations in *E. coli*, as well as its localization in *B. subtilis*, are affected by the ionophore carbonyl cyanide m-chlorophenylhydrazone [13]. This observation strongly suggests that MinD behaviour and, in turn, cell division, is strongly dependent on membrane potential. On the basis of this discovery, MinD mislocalization has been used as an indirect indicator or proxy for membrane depolarization [49–55]. There are caveats to this method, namely the fact that reduced levels of ATP may also lead to altered MinD function and localization, as MinD is an ATP-binding protein [55]. Moreover, delocalization of MinD may be also an indirect consequence of the effect of membrane potential changes on the localization of MreB, an actin homologue involved in cell wall synthesis [55]. It has been shown that MreB delocalization results in the perturbation of lipid homeostasis, which in turn affects the localization of other membrane proteins like MinD [56]. While these potential limitations need to be considered, the challenges in probing membrane potential dynamics with fluorescent dyes, especially dye exclusion by the outer membrane in Gram-negative bacteria [55,57–59], makes Min oscillations a tool well worth exploring. Membrane potential dynamics is essential in complex bacterial behaviour such as motility, antibiotic resistance, environmental sensing or electrical communication in biofilms, and is also key to understand the mechanism of action of antimicrobials [55,57,60,61].

## 5. Oscillations as a reporting tool in fluorescence microscopy

Min oscillations in *E. coli* are a classic example of collective dynamic behaviour of interacting components in a cell [62,63]. Spatio-temporal oscillations of this system provide the bacterium with information about its geometry, and thus showcase how large-scale properties of a cell can be assessed by processes at the molecular level [63]. Because of its relative simplicity, the Min system has been the subject of many theoretical models that recapitulate the oscillatory behaviour *in vivo* as well as in self-organized protein patterns on surfaces [62,64].

From the purely practical point of view of a live-cell fluorescence microscopy experiment, using oscillations as reporter of a biological parameter has advantages and limitations. The latter include slow data acquisition (ideally 2–3 full oscillation periods), which may be further limited by photobleaching or the long-term effect of immobilization. However, there are advantages to monitoring oscillations as opposed to changes in fluorescence intensity of a reporter. These advantages are similar to those provided by fluorescence lifetime imaging from the quantitative point of view, namely an independence of heterogeneities in the sample and imaging system, as well as in the fluorophore concentration [65]. This is particularly interesting for experiments in microfluidic systems or on non-uniform surfaces such as nanofabricated topographies mentioned above [48].

## 6. Conclusion and outlook

Min oscillations are a fascinating mechanism used by bacteria to find their middle. Beyond their biological role(s), they provide a relatively simple method to monitor the effect of environmental challenges on bacterial physiology in real time and at the single-cell level. In this review, we have discussed the original papers that put forward the idea of using Min oscillations as a reporting tool, at a time when the choice for fluorescent sensors was limited. More recently, our laboratory has been interested in reviving and expanding this method to understand the effect of other challenges on bacteria such as mechanical interaction or ROS. Further work is needed to establish more clearly the link between changes in the oscillation pattern and membrane potential dynamics, including quantification of the latter. However, the limitations in the use of membrane potential dyes in Gram-negative bacteria, due to their exclusion by the outer membrane, are a major motivation to further explore alternative methods to study and quantify changes in membrane potential.

In conclusion, Min oscillations are a useful addition to the toolbox of fluorescence microscopy methods to visualize stress responses in bacteria at the single-cell level and in real time [66,67]. In combination with other fluorescent reporters, it has the potential to provide full mechanistic understanding of the events that lead to bacterial cell death in different contexts.

## Data Availability

This article has no additional data.

## References

[1] Rowlett VW, Margolin W. 2013 The bacterial Min system. Curr. Biol. **23**, R553-R556. (10.1016/j.cub.2013.05.024) 23845239

[2] Rowlett VW, Margolin W. 2015 The Min system and other nucleoid-independent regulators of Z ring positioning. Front. Microbiol. **6**, 478. (10.3389/fmicb.2015.00478) 26029202 PMC4429545

[3] Merino-Salomón A, Babl L, Schwille P. 2021 Self-organized protein patterns: the MinCDE and ParABS systems. Curr. Opin. Cell Biol. **72**, 106-115. (10.1016/j.ceb.2021.07.001) 34399108

[4] Ramm B, Heermann T, Schwille P. 2019 The E. coli MinCDE system in the regulation of protein patterns and gradients. Cell Mol. Life Sci. **76**, 4245-4273. (10.1007/s00018-019-03218-x) 31317204 PMC6803595

[5] Wettmann L, Kruse K. 2018 The Min-protein oscillations in *Escherichia coli*: an example of self-organized cellular protein waves. Phil. Trans. R. Soc. B **373**, 20170111. (10.1098/rstb.2017.0111) 29632263 PMC5904297

[6] Taviti AC, Beuria TK. 2019 Bacterial Min proteins beyond the cell division. Crit. Rev. Microbiol. **45**, 22-32. (10.1080/1040841x.2018.1538932) 30526164

[7] Kelly C. 2011 Effect of antimicrobial agents on MinD protein oscillations in *Escherichia coli*. PhD thesis, University of Guelph, Guelph.

[8] Ortega IV, Torra J, Flors C. 2022 Min oscillations as real-time reporter of sublethal effects in photodynamic treatment of bacteria. ACS Infect. Dis. **8**, 86-90. (10.1021/acsinfecdis.1c00583) 35026951

[9] Del Valle A, Torra J, Bondia P, Tone CM, Pedraz P, Vadillo-Rodriguez V, Flors C. 2020 Mechanically induced bacterial death imaged in real time: a simultaneous nanoindentation and fluorescence microscopy study. ACS Appl. Mater. Interfaces **12**, 31 235-31 241. (10.1021/acsami.0c08184) 32476402

[10] Downing BPB, Rutenberg AD, Touhami A, Jericho M. 2009 Subcellular Min oscillations as a single-cell reporter of the action of polycations, protamine, and gentamicin on *Escherichia coli*. PLoS ONE **4**, e7285. (10.1371/journal.pone.0007285) 19789705 PMC2749335

[11] Touhami A, Jericho M, Rutenberg AD. 2006 Temperature dependence of MinD oscillation in *Escherichia coli*: running hot and fast. J. Bacteriol. **188**, 7661-7667. (10.1128/jb.00911-06) 16936014 PMC1636269

[12] Colville K, Tompkins N, Rutenberg AD, Jericho MH. 2010 Effects of poly(L-lysine) substrates on attached Escherichia coli bacteria. Langmuir **26**, 2639-2644. (10.1021/la902826n) 19761262

[13] Strahl H, Hamoen LW. 2010 Membrane potential is important for bacterial cell division. Proc. Natl Acad. Sci. USA **107**, 12 281-12 286. (10.1073/pnas.1005485107) PMC2901462 20566861

[14] de Boer PAJ, Crossley RE, Rothfield LI. 1989 A division inhibitor and a topological specificity Factor coded for by the minicell locus determine proper placement of the division septum in *E. coli*. Cell **56**, 641-649. (10.1016/0092-8674(89)90586-2) 2645057

[15] Rothfield L, Justice S, García-Lara J. 1999 Bacterial cell division. Annu. Rev. Genet. **33**, 423-448. (10.1146/annurev.genet.33.1.423) 10690414

[16] de Boer PAJ, Crossley RE, Hand AR, Rothfield LI. 1991 The MinD protein is a membrane ATPase required for the correct placement of the *Escherichia coli* division site. EMBO J. **10**, 4371-4380. (10.1002/j.1460-2075.1991.tb05015.x) 1836760 PMC453190

[17] Lutkenhaus J. 2002 Dynamic proteins in bacteria. Curr. Opin. Microbiol. **5**, 548-552. (10.1016/s1369-5274(02)00376-4) 12457696

[18] Yu XC, Margolin W. 1999 FtsZ ring clusters in min and partition mutants: role of both the Min system and the nucleoid in regulating FtsZ ring localization. Mol. Microbiol. **32**, 315-326. (10.1046/j.1365-2958.1999.01351.x) 10231488

[19] Hu Z, Lutkenhaus J. 1999 Topological regulation of cell division in Escherichia coli involves rapid pole to pole oscillation of the division inhibitor MinC under the control of MinD and MinE. Mol. Microbiol. **34**, 82-90. (10.1046/j.1365-2958.1999.01575.x) 10540287

[20] Lackner LL, Raskin DM, De Boer PAJ. 2003 ATP-dependent interactions between *Escherichia coli* Min proteins and the phospholipid membrane in vitro. J. Bacteriol. **185**, 735-749. (10.1128/jb.185.3.735-749.2003) 12533449 PMC142821

[21] Ramm B, Glock P, Mücksch J, Blumhardt P, García-Soriano DA, Heymann M, Schwille P. 2018 The MinDE system is a generic spatial cue for membrane protein distribution in vitro. Nat. Commun. **9**, 3942. (10.1038/s41467-018-06310-1) 30258191 PMC6158289

[22] Hu Z, Lutkenhaus J. 2003 A conserved sequence at the C-terminus of MinD is required for binding to the membrane and targeting MinC to the septum. Mol. Microbiol. **47**, 345-355. (10.1046/j.1365-2958.2003.03321.x) 12519187

[23] Hu Z, Saez C, Lutkenhaus J. 2003 Recruitment of MinC, an inhibitor of Z-ring formation, to the membrane in *Escherichia coli*: role of minD and minE. J. Bacteriol. **185**, 196-203. (10.1128/jb.185.1.196-203.2003) 12486056 PMC141945

[24] Park KT, Villar MT, Artigues A, Lutkenhaus J. 2017 MinE conformational dynamics regulate membrane binding, MinD interaction, and Min oscillation. Proc. Natl Acad. Sci. USA **114**, 7497-7504. (10.1073/pnas.1707385114) 28652337 PMC5530704

[25] Ayed SH, Cloutier AD, McLeod LJ, Foo ACY, Damry AM, Goto NK. 2017 Dissecting the role of conformational change and membrane binding by the bacterial cell division regulator MinE in the stimulation of MinD ATPase activity. J. Biol. Chem. **292**, 20 732-20 743. (10.1074/jbc.M117.805945) PMC5733608 29066619

[26] Loose M, Fischer-Friedrich E, Ries J, Kruse K, Schwille P. 2008 Spatial regulators for bacterial cell division self-organize into surface waves in vitro. Science **320**, 789-792. (10.1126/science.1154413) 18467587

[27] Meinhardt H, De Boer PAJ. 2001 Pattern formation in *Escherichia coli*: a model for the pole-to-pole oscillations of Min proteins and the localization of the division site. Proc. Natl Acad. Sci. USA **98**, 14 202-14 207. (10.1073/pnas.251216598) 11734639 PMC64659

[28] De Boer PAJ, Crossley RE, Rothfield LI. 1990 Central role for the *Escherichia coli* minC gene product in two different cell division-inhibition systems. Proc. Natl Acad. Sci. USA **87**, 1129-1133. (10.1073/pnas.87.3.1129) 2137246 PMC53424

[29] Margolin W. 2001 Bacterial cell division: A moving MinE sweeper boggles the MinD. Curr. Biol. **11**, R395-R398. (10.1016/s0960-9822(01)00217-2) 11378404

[30] Kretschmer S, Schwille P. 2016 Pattern formation on membranes and its role in bacterial cell division. Curr. Opin. Cell Biol. **38**, 52-59. (10.1016/j.ceb.2016.02.005) 26915065

[31] Raskin DM, De Boer PAJ. 1999 Rapid pole-to-pole oscillation of a protein required for directing division to the middle of *Escherichia coli*. Proc. Natl Acad. Sci. USA **96**, 4971-4976. (10.1073/pnas.96.9.4971) 10220403 PMC21801

[32] Palanisamy N, Öztürk MA, Akmeriç EB, Di Ventura B. 2020 C-terminal eYFP fusion impairs *Escherichia coli* MinE function. Open Biol. **10**, 200010. (10.1098/rsob.200010) 32456552 PMC7276532

[33] Huang KC, Meir Y, Wingreen NS. 2003 Dynamic structures in *Escherichia coli*: spontaneous formation of MinE rings and MinD polar zones. Proc. Natl Acad. Sci. USA **100**, 12 724-12 728. (10.1073/pnas.2135445100) PMC240685 14569005

[34] Halatek J, Frey E. 2012 Highly canalized MinD transfer and MinE sequestration explain the origin of robust MinCDE-protein dynamics. Cell Rep. **1**, 741-752. (10.1016/j.celrep.2012.04.005) 22813748

[35] Sabnis A et al. 2021 Colistin kills bacteria by targeting lipopolysaccharide in the cytoplasmic membrane. Elife **10**, e65836. (10.7554/eLife.65836) 33821795 PMC8096433

[36] Vaara M. 1992 Agents that increase the permeability of the outer membrane. Microbiol. Rev. **56**, 395-411. (10.1128/MR.56.3.395-411.1992) 1406489 PMC372877

[37] Jimenez-Banzo A, Nonell S, Hofkens J, Flors C. 2008 Singlet oxygen photosensitization by EGFP and its chromophore HBDI. Biophys. J. **94**, 168-172. (10.1529/biophysj.107.107128) 17766345 PMC2134865

[38] Lepe-Zuniga JL, Zigler JS, Gery I. 1987 Toxicity of light-exposed Hepes media. J. Immuno. Methods **103**, 145-146. (10.1016/0022-1759(87)90253-5) 3655381

[39] Wainwright M, Maisch T, Nonell S, Plaetzer K, Almeida A, Tegos GP, Hamblin MR. 2017 Photoantimicrobials—are we afraid of the light? Lancet Infect. Dis. **17**, e49-e55. (10.1016/s1473-3099(16)30268-7) 27884621 PMC5280084

[40] Cieplik F, Deng D, Crielaard W, Buchalla W, Hellwig E, Al-Ahmad A, Maisch T. 2018 Antimicrobial photodynamic therapy—what we know and what we don't. Crit. Rev. Microbiol. **44**, 571-589. (10.1080/1040841x.2018.1467876) 29749263

[41] Alves E, Faustino MAF, Neves MGPMS, Cunha A, Tome J, Almeida A. 2014 An insight on bacterial cellular targets of photodynamic inactivation. Future Med. Chem. **6**, 141-164. (10.4155/fmc.13.211) 24467241

[42] Del Valle García A. 2020 Simultaneous fluorescence and atomic force microscopy to study mechanically-induced bacterial death in real time. PhD Thesis, Universidad Autónoma de Madrid, Madrid.

[43] Jenkins J, Mantell J, Neal C, Gholinia A, Verkade P, Nobbs AH, Su B. 2020 Antibacterial effects of nanopillar surfaces are mediated by cell impedance, penetration and induction of oxidative stress. Nature Commun **11**, 1626. (10.1038/s41467-020-15471-x) 32242015 PMC7118135

[44] Zhao S et al. 2022 Programmed death of injured Pseudomonas aeruginosa on mechano bactericidal surfaces. Nano Lett **22**, 1129-1137. (10.1021/acs.nanolett.1c04243) 35040647

[45] Ishak MI, Jenkins J, Kulkarni S, Keller TF, Briscoe WH, Nobbs AH, Su B. 2021 Insights into complex nanopillar-bacteria interactions: Roles of nanotopography and bacterial surface proteins. J Colloid Interface Sci **604**, 91-103. (10.1016/j.jcis.2021.06.173) 34265695

[46] Rizzello L, Sorce B, Sabella S, Vecchio G, Galeone A, Brunetti V, Cingolani R, Pompa PP. 2011 Impact of nanoscale topography on genomics and proteomics of adherent bacteria. ACS Nano **5**, 1865-1876. (10.1021/nn102692m) 21344880

[47] Liu S, Keong Ng A, Xu R, Wei J, Tan CM, Yang Y, Chen Y. 2010 Antibacterial action of dispersed single-walled carbon nanotubes on *Escherichia coli* and *Bacillus subtilis* investigated by atomic force microscopy. Nanoscale **2**, 2744-2750. (10.1039/C0NR00441C) 20877897

[48] Viela F, Ortega IV, Hernández JJ, Rodríguez I, Moreno-Da Silva S, López-Moreno A, Pérez EM, Flors C. 2023 Real-time imaging of the mechano-bactericidal action of colloidal nanomaterials and nanostructured topographies. Small Sci. **3**, 2300002. (10.1002/smsc.202300002) PMC11935832 40213324

[49] Chimerel C, Field CM, Piñero-Fernandez S, Keyser UF, Summers DK. 2012 Indole prevents Escherichia coli cell division by modulating membrane potential. Biochim. Biophys. Acta. Biomemb. **1818**, 1590-1594. (10.1016/j.bbamem.2012.02.022) PMC3793866 22387460

[50] Eun YJ, Foss MH, Kiekebusch D, Pauw DA, Westler WM, Thanbichler M, Weibel DB. 2012 DCAP: a broad-spectrum antibiotic that targets the cytoplasmic membrane of bacteria. JACS **134**, 11 322-11 325. (10.1021/ja302542j) PMC3516701 22741745

[51] Foss MH, Eun YJ, Grove CI, Pauw DA, Sorto NA, Rensvold JW, Pagliarini DJ, Shaw JT, Weibel DB. 2013 Inhibitors of bacterial tubulin target bacterial membranes in vivo. Med. Chem. Commun. **4**, 112-119. (10.1039/C2MD20127E) PMC3607388 23539337

[52] Wenzel M, Kohl B, Münch D, Raatschen N, Albada HB, Hamoen L, Metzler-Nolte N, Sahl HG, Bandow JE. 2012 Proteomic response of *Bacillus subtilis* to lantibiotics reflects differences in interaction with the cytoplasmic membrane. Antimicrob. Agents Chemother. **56**, 5749-5757. (10.1128/aac.01380-12) 22926563 PMC3486579

[53] Wenzel M et al. 2013 Analysis of the mechanism of action of potent antibacterial hetero-tri-organometallic compounds: a structurally new class of antibiotics. ACS Chem. Biol. **8**, 1442-1450. (10.1021/cb4000844) 23578171

[54] Wenzel M, Schriek P, Prochnow P, Albada HB, Metzler-Nolte N, Bandow JE. 2016 Influence of lipidation on the mode of action of a small RW-rich antimicrobial peptide. Biochim. Biophys. Acta Biomemb. **1858**, 1004-1011. (10.1016/j.bbamem.2015.11.009) 26603779

[55] te Winkel JD, Gray DA, Seistrup KH, Hamoen LW, Strahl H. 2016 Analysis of antimicrobial-triggered membrane depolarization using voltage sensitive dyes. Front. Cell Dev. Biol. **4**, 29. (10.3389/fcell.2016.00029) 27148531 PMC4829611

[56] Strahl H, Bürmann F, Hamoen LW. 2014 The actin homologue MreB organizes the bacterial cell membrane. Nat. Commun. **5**, 3442. (10.1038/ncomms4442) 24603761 PMC3955808

[57] Benarroch JM, Asally M. 2020 The microbiologist's guide to membrane potential dynamics. Trends Microbiol. **28**, 304-314. (10.1016/J.TIM.2019.12.008) 31952908

[58] Buttress JA, Halte M, te Winkel JD, Erhardt M, Popp PF, Strahl H. 2022 A guide for membrane potential measurements in Gram-negative bacteria using voltage-sensitive dyes. Microbiology **168**, 001227. (10.1099/mic.0.001227) 36165741

[59] Hudson MA, Siegele DA, Lockless SW. 2020 Use of a fluorescence-based assay to measure *Escherichia coli* membrane potential changes in high throughput. Antimicrob. Agents Chemother. **64**, e00910-e00920. (10.1128/aac.00910-20) 32631824 PMC7449193

[60] Schäfer A-B, Wenzel M. 2020 A how-to guide for mode of action analysis of antimicrobial peptides. Front. Cell Infect Microbiol. **10**, 1-27. (10.3389/fcimb.2020.540898) 33194788 PMC7604286

[61] Jin X et al. 2023 Sensitive bacterial Vm sensors revealed the excitability of bacterial Vm and its role in antibiotic tolerance. Proc. Natl Acad. Sci. USA **120**, e2208348120. (10.1073/PNAS.2208348120) 36623202 PMC9934018

[62] Cao Y, Lopatkin A, You L. 2016 Elements of biological oscillations in time and space. Nat. Struct. Mol. Biol. **23**, 1030-1034. (10.1038/nsmb.3320) 27922613

[63] Kruse K, Jülicher F. 2005 Oscillations in cell biology. Curr. Opin. Cell Biol. **17**, 20-26. (10.1016/j.ceb.2004.12.007) 15661515

[64] Lenz P, Søgaard-Andersen L. 2011 Temporal and spatial oscillations in bacteria. Nat. Rev. Microbiol. **9**, 565-577. (10.1038/nrmicro2612) 21760621

[65] Becker W. 2012 Fluorescence lifetime imaging—techniques and applications. J. Micros. **247**, 119-136. (10.1111/j.1365-2818.2012.03618.x) 22621335

[66] Lagage V, Uphoff S. 2020 Pulses and delays, anticipation and memory: seeing bacterial stress responses from a single-cell perspective. FEMS Microbiol. Rev. **44**, 565-571. (10.1093/femsre/fuaa022) 32556120

[67] Choi H, Rangarajan N, Weisshaar JC. 2016 Lights, camera, action! Antimicrobial peptide mechanisms imaged in space and time. Trends Microbiol. **24**, 111-122. (10.1016/j.tim.2015.11.004) 26691950 PMC4733415

